# Early-onset lung cancer in Asia: a narrative review

**DOI:** 10.3389/fonc.2025.1631443

**Published:** 2025-08-04

**Authors:** Roselle B. De Guzman

**Affiliations:** Department of Medicine, Manila Central University-FDT Medical Foundation Hospital, Caloocan, Metro Manila, Philippines

**Keywords:** lung cancer, familial lung cancer, early-onset lung cancer, young adults, Asia

## Abstract

**Introduction:**

The incidence of early-onset lung cancer (EOLC), often defined as those that occur in adults under the age of 50, is increasing globally. Asia accounted for almost 76% of EOLC cases worldwide. This presents a challenge given previous limited studies and the socioeconomic implications.

**Methods:**

References were identified through a PubMed, Scopus and Web of science search for relevant articles published in 2000 to April 2025 using the terms ‘lung carcinoma or cancer’, ‘early-onset’ or ‘young adults’, and ‘Asia’.

**Results:**

Early-onset lung cancers are more common in female, primarily presenting as adenocarcinoma, and tends to be diagnosed at an advanced stage. There is a high prevalence of potentially targetable genomic alterations: 30-56.3% EGFR mutations and 16.1-50% ALK rearrangements. Comparative analyses show higher prevalence of actionable genetic alterations ROS1, and RET fusions and ERBB2 mutations compared to older patients. Air pollution is a significant risk factor for lung cancer in Asia. MUC16, a transmembrane glycoprotein, is overexpressed in lung cancer, particularly in patients exposed to indoor air pollution. Patients with EOLC exhibit impaired cell-mediated immunity with reduced T cell infiltration. This suggests a potentially limited response to immune checkpoint inhibitor therapies in this patient group.

**Conclusion:**

The incidence of EOLC is increasing in Asia. This accounts for 75.9% of global cases. The research conducted in the region are mostly retrospective and, majority are single-institution studies. Due to its unique clinical and molecular features, EOLC requires dedicated research efforts and tailored interventions.

## 1 Introduction

Lung cancer remains the most common cause of cancer-related deaths globally. It represents 12.4% of all cancer cases and 18.7% of cancer mortality (1). Risk factors of lung cancer include tobacco smoking, environmental tobacco smoke or second-hand smoking, occupational exposures, air pollution, previous respiratory diseases, advanced age and genetic susceptibility (2–4). The occurrence of lung cancer increases significantly in individuals over the age of 50. The studies that have been conducted focused on the risk factors in these age group and in older population. There is inadequate information on early-onset lung cancer (EOLC), often defined as those that occur in adults under the age of 50 (5). Assessing risk factors, distinct clinicopathologic features and behavior, tumor molecular characteristics, and management outcomes for early-onset lung cancer present a challenge given previous limited studies and socioeconomic implications. This article reviews the clinical and molecular characteristics of EOLC in Asia based on retrospective analyses, research studies, and reviews currently available in the scientific literature.

## 2 Methods

### 2.1 Study design, search strategy and selection

References for this Review were identified through a comprehensive search of PubMed, Scopus and Web of Science for relevant publications from 2000 until April 2025. The search was limited to articles that were published in English language. The search terms used included “lung carcinoma or lung cancer”, “early-onset” or “young adults”, and “Asia”. The search covered a wide range of publication types, including original research articles, reviews, editorials, cohort and cross-sectional studies, as well as letters to the editor. Additional articles were sourced from the authors’ personal archives and by reviewing the reference lists of pertinent studies to identify papers not retrieved through the initial electronic search. The final list of references was selected based on originality, recency, and relevance to the objectives and scope of this review. The guidelines of the Preferred Reporting Items for Systematic Reviews and Meta-Analyses (PRISMA) (6) were followed to systematically search the literature to identify studies relevant to this review. Duplicate papers, papers not written in English, those containing non-human and aged 65+ years were manually removed through the study screening process.

### 2.2 Results

**Figure 1:**
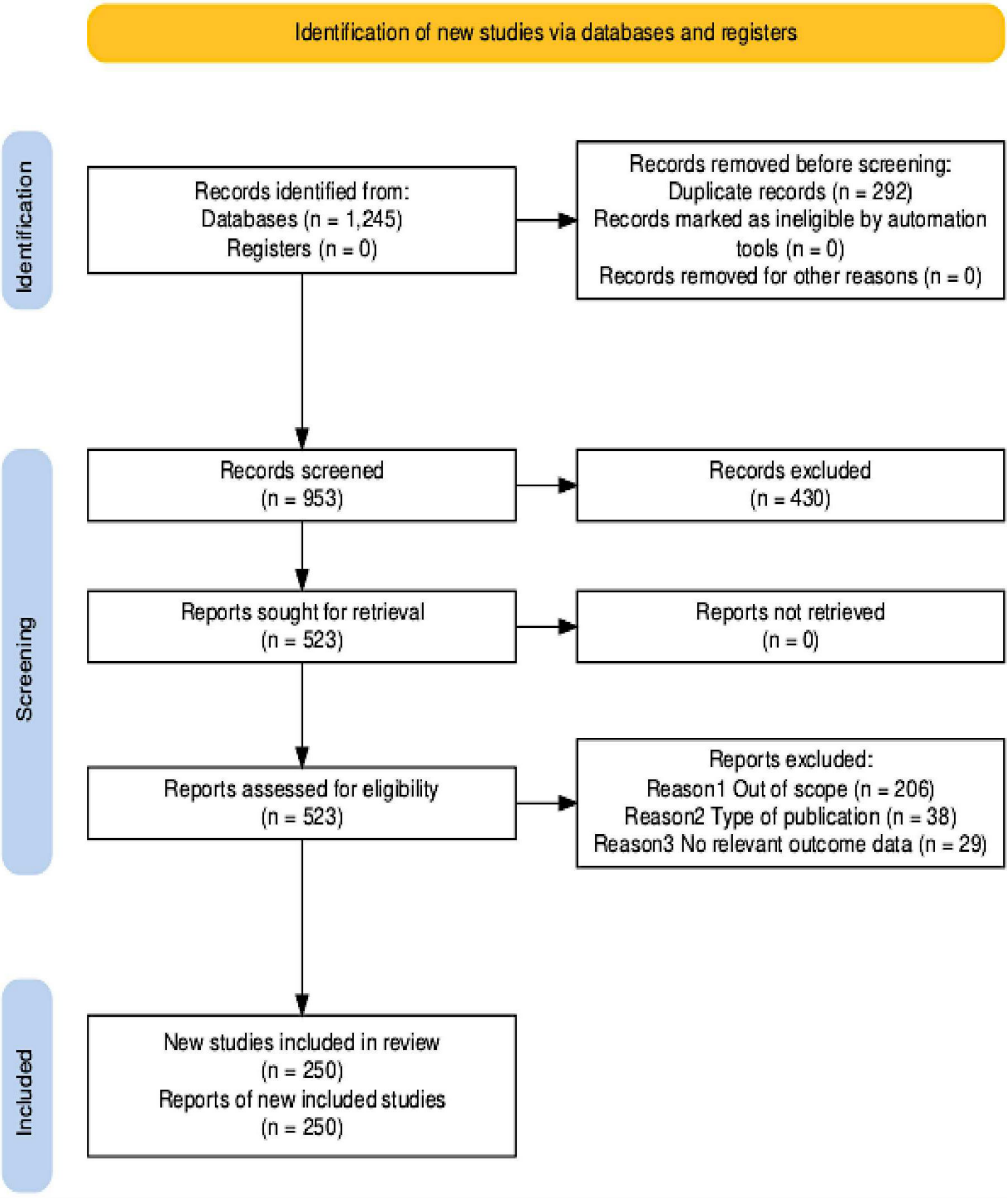
PRISMA flow diagram illustrating article selection strategy.

The PRISMA flow diagram (**Figure 1**) shows the study selection process. The search strategy initially identified 1,196 records. The included articles were published between 2000 and April 2025. The subsequent process involved several stages to refine the initial search results. Before screening, 243 duplicate records were systematically removed. This left 953 unique records. The final list of references that included 523 articles was selected based on originality, recency, and relevance to the objectives and scope of this review.

## 3 Epidemiology

**Figure 2:**
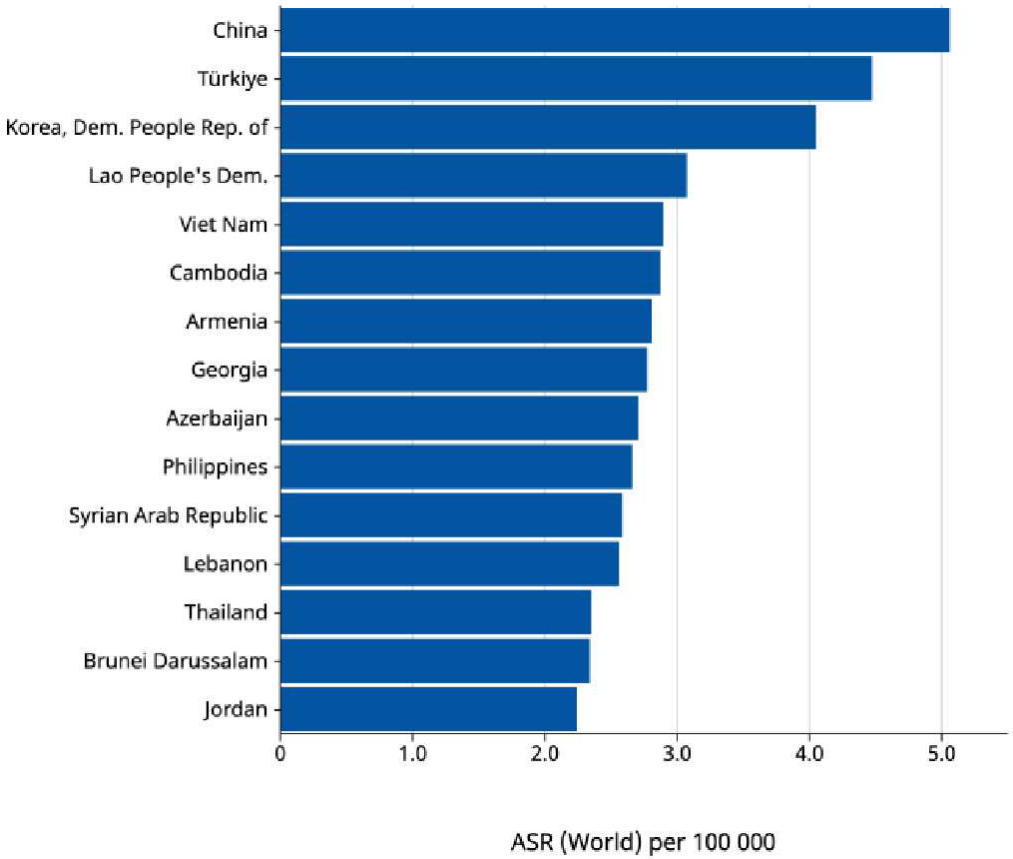
Age-standardized rate (World) per 100 000, Indidence, Both sexes, age [0-49], in 2022. Trachea, bronchus and lung cancer in Asia (Top 15 countries) Cancer TODAY, AIRC https://gco.iarc.who.int Globocan 2022.

**Figure 3:**
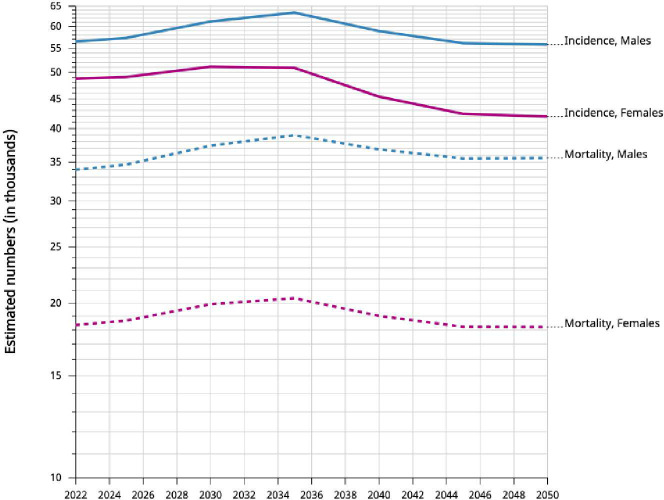
Incidence and mortality. Estimated numbers from 2022 to 2050, Males and Females, age [0-49]. Trachea, bronchus and lung. Asia Cancer TODAY, IARC https://gco.iarc.who.int Globocan 2022.

The incidence of early-onset cancers is rising globally (7). In 2019, the incidence exceeded 3.26 million cases (7). This was 79.1% increase since 1990 (6). Asia accounted for almost 76% of EOLC cases globally (1). China, Turkiye and Korea have the highest incidence in the region based on age-standardized rate per 100,000 (8; **Figure 2**). China, India and Indonesia have the highest number of EOLC cases in 2022 (8). China contributes 64.1% of these cases (8). EOLC incidence and mortality are expected to increase, with 110,000 new cases by year 2035 (9) (**Figure 3**).

Based on current data and projections for the next years, there is the need for research studies and targeted interventions to address this significant public health concern.

## 4 Risk factors

A study in Korea by Kang, et al. (10) is the largest cohort to date evaluating the health behaviors, socioeconomic factors, and comorbidities associated with lung cancer risk in individuals under the age of 40. The risk of developing lung cancer was observed to increase with advancing age, and females were found to be at greater risk compared to males (10). While current smoking was significantly associated with a heightened risk, former smoking did not show the same effect. Additionally, metabolic syndrome was linked to an increased likelihood of lung cancer. In contrast, light alcohol intake (less than 10 g/day) seemed to offer a protective benefit, though this effect was not seen with higher levels of consumption (10 g/day or more) (10). Factors such as higher income, regular physical activity, and obesity were associated with a lower risk of developing lung cancer (10).

### 4.1 Air pollution

Air pollution is a significant risk factor for lung cancer in Asia (11–14). Both ambient air pollution, specifically fine particulate matter (PM2.5), and household air pollution from the use of solid fuels like coal have been associated with increased lung cancer risk in various Asian populations, including those in China, Korea, and Taiwan (15–17). The International Agency for Research on Cancer (IARC) has classified outdoor air pollution and particulate matter as Group 1 carcinogens (18, 19).

A study from North China (12) provides specific insights on EOLC and air pollution. This large multicenter case–control study involved over 14,000 subjects. It identified being younger than 50 years old as a risk factor for lung cancer overall (12). More specifically, in areas categorized as heavily polluted, being male, consistent alcohol consumption, a family history of cancer, having ever smoked, and having quit smoking were all identified as risk factors for lung cancer (12). Being younger than 40 years old, having a family history of cancer, and smoking or having smoked in the past were found to be higher risk factors in the heavily polluted areas (12). This finding suggests a potential interaction where heavy air pollution exposure may contribute to earlier onset of the disease, particularly when combined with genetic predisposition or family history and smoking history. The study also highlighted that PM2.5 was the main factor affecting the occurrence of lung cancer in this North China cohort (12).

The higher levels of air pollution (20) is one possible reason to explain the higher familial risk of lung cancer in Asia. The traditional indoor cooking methods by burning coal or solid fuel are still practiced in certain rural areas in China (21) and in some countries in Southeast Asia. Xuanwei county has the highest lung cancer rates in China which was attributed to household air pollution from cooking and heating (22, 23).

### 4.2 Familial lung cancer

Earlier research on the familial risk of lung cancer has shown that the risk increases when there is a history of early-onset lung cancer in the family, affected first-degree relatives (FDRs), as well as multiple affected family members (24, 25). Individuals with a family history of lung cancer among FDRs have approximately 50% higher risk of lung cancer than those without a family history (24).

Characteristics of FLC in Yunnan-Guizhou, China found more individuals under age 50, showed two peaks, i.e. 46 vs. 58 years (26). This suggested genetic or environmental potential risk factors. While not statistically significant, the FLC group showed higher proportions of females, adenocarcinoma histology, stage IV disease, and metastasis to other organs, suggesting a more aggressive cancer (26). A significant difference was observed in tumor location, with FLC patients more commonly having cancer in the left lung (26).

A systematic review and meta-analysis done by Ang et al. (27) found that sex, smoking status, type of FDR, early onset of lung cancer in affected relatives, and having at least one affected relative was significantly associated with increased familial risk of lung cancer. Familial risk of lung cancer was found to be greater in Asian populations compared to those in Western regions, in younger individuals compared to older ones, and in those with two or more affected relatives as opposed to those with only one (27). Asians were observed to have a higher familial risk of developing squamous cell carcinoma compared to Western counterparts. Higher familial lung cancer risk among younger individuals suggests a genetic component favoring a tumor suppressor model with cellularly recessive effects that are commonly found in familial cancers such as breast cancer (28). Evidence of Mendelian codominant inheritance caused by a rare major autosomal gene for the pathogenesis of early onset familial risk of lung cancer has been previously reported (29).

Familial risk of lung cancer is complex. It involves both genetic and the shared environmental factors including environmental tobacco smoke, air pollution, and geographical region among family members (30). The effects of smoking and smoking behavior cannot fully explain and account for familial risk of lung cancer. Genetic factors, including polymorphisms in DNA repair genes, impaired DNA damage, and defective DNA repair mechanisms, may contribute to the susceptibility of familial lung cancer in both never-smokers and ever-smokers (31–33). Germline genetic mutation of epidermal growth factor receptor (*EGFR*) T790 M were identified in families with high density of lung cancer cases (34, 35).

Individuals with a family history of early-onset lung cancer have a significantly increased risk of developing the disease, whereas this link was not observed in those with relatives diagnosed at a later age. A rare major autosomal gene could play a role in early-onset lung cancer (36). A prior study conducted in Anhui, China, reported that female relatives, particularly mothers, had a higher risk of lung cancer compared to male relatives (37). The control for household exposure to tobacco smoke did not negate the finding that females with family history of lung cancer were more susceptible to lung cancer risk than their male counterparts (33).

### 4.3 Pulmonary tuberculosis

History of TB infection has been considered as a significant independent risk factor for lung cancer (38). Hwang SY, et al. did a comprehensive systematic review and meta-analysis (39) that showed a statistically significant association between a prior TB diagnosis and subsequent lung cancer. This association was found to be more pronounced in younger patients diagnosed with pulmonary TB (39). The link is particularly evident in regions with a high or upper-middle burden of TB, such as in East Asia and the Pacific (39). The increased risk is independent of smoking status. Several mechanisms have been proposed explaining this correlation, including TB-induced chronic inflammation, potential immunosuppressive effects, and the capacity of *Mycobacterium tuberculosis* to induce cellular DNA damage (40–42). The effective control and treatment of TB, including latent TB infection, may play a role in mitigating the risk of future lung cancer development.

### 4.4 Reproductive and hormonal factors

The influence of reproductive and hormonal factors on lung cancer in women suggests a direct hormonal etiology (43, 44). In a pooled analysis of over 308,000 Asian females done by Yin X, et al. revealed that parous women, especially those with one or two children, had a lower risk of incidence as well as mortality from lung cancer when compared to nulliparous women (45). This was found to be even greater among ever-smokers than never-smokers (45). Additionally, an older age at first delivery was linked to a reduced risk of lung cancer incidence and mortality (45). A comprehensive meta-analysis (46) supported these observations. In addition to higher parity and older age at first delivery, longer menstrual cycle length was also significantly associated with a reduced risk of lung cancer (46). These protective effects were notably observed among Asian women (46). Conversely, ever-use of hormone replacements and non-natural menopause increased the likelihood of lung cancer (46). These are often attributed to the potential effects of estrogen on lung cancer proliferation and growth (47). Estrogen receptors are expressed in both normal and cancerous lung tissues, allowing estrogen to directly stimulate gene transcription and transactivate growth factor signaling pathways, particularly the epidermal growth factor receptor (EGFR) pathway (48). The factors associated with lower estrogen levels have been correlated with a decreased lung cancer risk (49). Given the prevalence of EGFR mutations in adenocarcinoma among East Asian females who are never-smokers, it is hypothesized that higher parity might inversely affect lung cancer risk by inhibiting EGFR activation or mutation (50).

## 5 Clinical presentations

Studies from China, Japan, and India (51–55) offer valuable insights into the clinical presentation and characteristics of early-onset lung cancer in Asia. In a large Chinese cohort (51) early-onset non-small cell lung cancer (NSCLC) represented 5.27% of all lung cancer cases among individuals aged 15 to 45 years. Studies indicate that this younger population often presents with adenocarcinoma as the predominant histological subtype, reported in 85.7% of patients in India (55) and a high proportion (45%) in a retrospective study reviewing patients aged 18–35 years (51). Furthermore, a significant number of these patients are never smokers (44-79%) (54, 55). Gender distribution appears notable, with one large Chinese study observing a higher female-to-male ratio (2.55 vs 1.19) in early-onset lung adenocarcinoma patients compared to later-onset cases. Diagnosis often occurs at late-stage disease, with 49.21% being stage IV at diagnosis (51). Metastasis occurs frequently, with the pleura being the most common site (38.71%), followed by the bone (35.48%) and lung (25.81%), and most cases (68%) involving metastasis to a single organ (51).

## 6 Molecular etiology and genomic alterations

Young patients with lung cancer had a higher frequency of genomic alterations (51, 56). The first study to characterize the genomic alterations of lung adenocarcinoma in young never-smokers aged 45 years and younger through whole genome sequencing was done by Luo, et al. (57). Although the sample size was small, there was a high prevalence (63.9%) of potentially targetable genomic alterations in the cohort (57). Several analyses on patients with adenocarcinoma, aged ≤ 40 years found 30-56.3% harbored EGFR mutations, 16.1-50% had *ALK* rearrangements, and *ROS1* in 7% (54, 55, 58–62). Hou et al. (60) reported that in patients with lung adenocarcinoma aged 45 years or younger, there was a higher occurrence of genetic alterations in human epidermal growth factor receptor 2 (HER2) and ALK, along with concurrent EGFR/TP53 mutations. In contrast, the prevalence of EGFR exon 20 mutations, KRAS, and serine/threonine kinase 11 (STK11) mutations was lower compared to patients older than 45 years. A retrospective Japanese study of 1746 consecutive patients diagnosed with adenocarcinoma identified 81 who were aged 40 years or younger at diagnosis (54). The specific alterations that were observed in the most common actionable targets included the following: *HER2* mutations (5.7%), BRAF (3.4%), v-Ki-ras2 Kirsten rat sarcoma viral oncogene homolog (KRAS) mutations (2-3.4%), Ret proto-oncogene (RET) (1.1%), CDKN2A (1.1%), HER2 amplification (1.1%), HRAS (1.1%), and MAP2K1 (1.1%) (54).

Most of the available research studies in Asia were on comparative analyses on the genomic alteration between early-onset or younger patients and elder or late-onset lung adenocarcinoma (52, 63). One analysis (64) reveals more frequent *ERBB2* mutations and *ALK*-rearrangement in younger group. A study analyzing 7,858 lung cancer samples through targeted-gene sequencing examined the genomic differences between younger (≤ 45 years) and older (> 45 years) patients (64). The age-dependent trend analysis for genomic alterations showed increases in tumor mutation burden and changes in several genes as age advanced, including KRAS, MET, PIK3CA, CDKN2A, and MDM2. Conversely, the frequencies of ALK, ROS1, and RET fusions, as well as ERBB2 mutations, were found to decrease with age (64).

Comparisons of young and old patients found that young patients were characterized by a higher prevalence of *ALK*, *ROS1*and *RET* fusions, and *ERBB2* exon-20 insertions and *EGFR* exon-19 deletions (64). Approximately 88% of younger patients were identified as having at least one actionable genetic mutation. Somatic interaction analysis suggested that younger individuals with *EGFR*-positive tumors were more frequently associated with additional mutations in genes such as *PIK3CA, MET, TP53, and RB1* compared to their older counterparts (64).

The unique genomic landscape of EOLC carries important clinical implications. The use of next-generation sequencing for patients is essential to support individualized treatment approaches. For those with driver gene mutations, the use of targeted therapies is crucial for effective disease management and improved outcomes.

## 7 Gene expression and immune landscape

MUC16, a large transmembrane glycoprotein (65–67), has been shown to be associated with enhanced cancer cell growth, metastasis and chemo resistance (68–70). MUC16 could be a therapeutic target for patients (71). MUC16 expression and its clinical significance has been investigated in patients with FLC and affected by indoor air pollution (72). The study revealed that there was a significant association between MUC16 overexpression and FLC (P<0.05), indoor air pollution (P<0.01) and later disease stage (P<0.01) (72). It was also noted that there were more metastasis cases when MUC16 was up regulated (18.1% vs. 10.3%). Additionally, patients with more MUC16 up-regulation were noted to have a lower white blood cell (72). This could play a role in immune regulation. Findings from this study provide useful information on the interaction between environmental carcinogens and genetic background.

A key characteristic of EOLC is deficiency in cell-mediated immunity of younger patients as demonstrated by transcriptomic and immunological analyses (63). There were changes in cellular metabolism and immune-related genes. There were also reduced immune cells infiltration (49). Notably, there is a significant decrease in CD4+, CD8+, and CD3+ T cells at both tumor sites and tumor/non-tumor interface zones (63). This finding indicates an immunosuppressive tumor microenvironment. Although current treatments for NSCLC often rely on immune checkpoint inhibitors to enhance T cell-mediated anti-tumor responses (73), these findings suggest that such therapies may be less effective in EOLC.

## 8 Considerations for future perspectives

Despite growing interest in EOLC, most existing research remains limited to single-institution studies, with a significant proportion of Asian data derived from small, retrospective cohorts. Consequently, there is a lack of comprehensive, nationwide or regional data on clinical and survival outcomes. There is a need to better understand the disease’s underlying biology. The potential contribution of dietary patterns and environmental exposures, including air pollution, remains poorly elucidated and warrants further investigation. Additionally, there is a notable scarcity of clinical trials specifically designed for EOLC, limiting evidence-based approaches to management and care in this population.

Current lung cancer screening guidelines are primarily tailored to older adults (≥50 years) with significant smoking histories, thereby excluding a large subset of younger patients who do not meet traditional high-risk criteria. This exclusion may contribute to diagnostic delays and worse prognostic outcomes in patients with EOLC. As screening models may not effectively capture the distinct risk profile of this group, there is a pressing need to refine risk stratification strategies and incorporate novel, efficient biomarkers for earlier detection. Furthermore, future studies should aim to validate emerging molecular findings in larger, more diverse patient populations to better define the genomic landscape of EOLC. These efforts are crucial to developing targeted prevention strategies, as well as in optimizing therapeutic interventions for this underrepresented and often overlooked patient population.

## 9 Limitations

A limitation of this narrative review is that the article selection process was conducted by a single reviewer. While this approach ensured consistency in the selection criteria, it carries a risk of selection bias, and may have inadvertently led to overlook other relevant studies. Literature search strategy relied primarily on keywords without the use of Medical Subject Headings (MeSH) terms that may have limited the comprehensiveness of the search.

## 10 Conclusions

The rising incidence of EOLC, particularly in Asia, which accounts for over 75% of global cases, signals a growing public health concern that remains poorly understood. Given its distinct clinical and molecular features, EOLC demands dedicated research, tailored screening strategies, and targeted interventions. This is particularly significant due to personal, familial, and societal impacts associated with cancers affecting individuals in their most productive years. A better and deeper understanding of the epidemiology, risk factors, and the biology of EOLC will be critical to inform public health strategies and improve outcomes.

## References

[1] Bray F Laversanne M Sung H Siegel RL Ferlay J Jemal A. Global cancer statistics 2022: GLOBOCAN estimates of incidence and mortality worldwide for 36 cancers in 185 countries. CA Cancer J Clin. (2024) 74:229–2631. doi: 10.3322/caac.21834, PMID: 38572751

[2] Kanwal M Ding XJ Cao Y. Familial risk for lung cancer. Oncol Lett. (2017) 13:535–5421. doi: 10.3892/ol.2016.5518, PMID: 28356926 PMC5351216

[3] Malhotra J Malvezzi M Negri E La Vecchia C Boffetta P. Risk factors for lung cancer worldwide. Eur Respir J. (2016) 48:889–9021. doi: 10.1183/13993003.00359-2016, PMID: 27174888

[4] Seow WJ Matsuo K Hsiung CA Shiraishi K Song M NamKim H. Association between GWAS-identified lung adenocarcinoma susceptibility loci and EGFR mutations in never-smoking Asian women, and comparison with findings from Western populations. Hum Mol Genet. (2017) 26:454–4651. doi: 10.1093/hmg/ddw414, PMID: 28025329 PMC5856088

[5] Ledford H. Why are so many young people getting cancer? What the data say. Nature. (2024) 627:258–2601. doi: 10.1038/d41586-024-00720-6, PMID: 38480960

[6] Haddaway NR Page MJ Pritchard CC McGuinness LA. PRISMA2020: An R package and Shiny app for producing PRISMA 2020-compliant flow diagrams, with interactivity for optimised digital transparency and Open Synthesis. Campbell Systematic Reviews. (2022) 18:. doi: 10.1002/cl2.1230, PMID: 36911350 PMC8958186

[7] Zhao J Xu L Sun J Song M Wang L Yuan S. Global trends in incidence, death, burden and risk factors of early-onset cancer from 1990 to 2019. BMJ Oncol. (2023) 2:e0000491. doi: 10.1136/bmjonc-2023-000049, PMID: 39886513 PMC11235000

[8] International Agency for Research on Cancer. Cancer TODAY. Available online at: https://gco.iarc.fr/today/en1 (Accessed April 27, 2025).

[9] International Agency for Research on Cancer. Cancer TOMORROW. Available online at: https://gco.iarc.fr/tomorrow/en1 (Accessed April 27, 2025).

[10] Kang J Kim T Han KD Jung JH Jeong SM Yeo YH. Risk factors for early-onset lung cancer in Korea: analysis of a nationally representative population-based cohort. Epidemiol Health. (2023) 45:e20231011. doi: 10.4178/epih.e2023101, PMID: 38037323 PMC10876445

[11] Guo H Chang Z Wu J Li W. Air pollution and lung cancer incidence in China: who are faced with a greater effect? Environ Int. (2019) 132:1050771. doi: 10.1016/j.envint.2019.105077, PMID: 31415963

[12] Li D Shi J Liang D Ren M He Y. Lung cancer risk and exposure to air pollution: a multicenter North China case-control study involving 14604 subjects. BMC Pulm Med. (2023) 23:1821. doi: 10.1186/s12890-023-02480-x, PMID: 37226220 PMC10210434

[13] Tsai SS Chiu YW Weng YH Yang CY. Association between fine particulate air pollution and the risk of death from lung cancer in Taiwan. J Toxicol Environ Health A. (2022) 85:431–4381. doi: 10.1080/15287394.2022.2040672, PMID: 35216542

[14] Zhang Z Zhu D Cui B Ding R Shi X He P. Association between particulate matter air pollution and lung cancer. Thorax. (2020) 75:85–871. doi: 10.1136/thoraxjnl-2019-213722, PMID: 31727788

[15] Hosgood HD 3rd Song M Hsiung CA Yin Z Shu XO Wang Z. Interactions between household air pollution and GWAS-identified lung cancer susceptibility markers in the Female Lung Cancer Consortium in Asia (FLCCA). Hum Genet. (2015) 134:333–3411. doi: 10.1007/s00439-014-1528-z, PMID: 25566987 PMC5537621

[16] Zhang H Liu C Wang S Wang Q Feng X Jiang H. Proteogenomic analysis of air-pollution-associated lung cancer reveals prevention and therapeutic opportunities. eLife. (2024) 13:RP954531. doi: 10.7554/eLife.95453.3, PMID: 39432560 PMC11493407

[17] Ji C Lv J Zhang J Zhu M Yu C Ma H. Household air pollution and risk of incident lung cancer in urban China: A prospective cohort study. Int J Cancer. (2023) 153(9):1592–601. doi: 10.1002/ijc.34646, PMID: 37403464

[18] IARC Working Group on the Evaluation of Carcinogenic Risks to Humans. Outdoor Air Pollution Vol. 109. . Lyon, France: International Agency for Research on Cancer (2016) p. 9–4441. PMC7682275 29905447

[19] Loomis D Grosse Y Lauby-Secretan B El Ghissassi F Bouvard V Benbrahim-Tallaa L. The carcinogenicity of outdoor air pollution. Lancet Oncol. (2013) 14:1262–12631. doi: 10.1016/S1470-2045(13)70487-X, PMID: 25035875

[20] Xing DF Xu CD Liao XY Xing TY Cheng SP Hu MG. Spatial association between outdoor air pollution and lung cancer incidence in China. BMC Public Health. (2019) 19:13772. doi: 10.1186/s12889-019-7740-y, PMID: 31655581 PMC6815434

[21] Mu L Liu L Niu R Zhao B Shi J Li Y. Indoor air pollution and risk of lung cancer among Chinese female non-smokers. Cancer Causes Control. (2013) 24:439–4502. doi: 10.1007/s10552-012-0130-8, PMID: 23314675 PMC3574203

[22] Cao Y Gao H. Prevalence and causes of air pollution and lung cancer in Xuanwei City and Fuyuan County. Yunnan Province China. (2012) 6(2):217–20. doi: 10.1007/s11684-012-0192-8, PMID: 22573219

[23] Seow WJ Hu W Vermeulen R Hosgood HD Downward GS Chapman RS. Household air pollution and lung cancer in China: A review of studies in Xuanwei. Chin J Cancer. (2014) 33:471–4752. doi: 10.5732/cjc.014.10132, PMID: 25223911 PMC4198749

[24] Bailey-Wilson JE Amos CI Pinney SM Petersen GM De Andrade M Wiest JS. A major lung cancer susceptibility locus maps to chromosome 6q23-25. Am J Hum Genet. (2004) 75:460–4742. doi: 10.1086/423857, PMID: 15272417 PMC1182024

[25] Coté ML Liu M Bonassi S Neri M Schwartz AG Christiani DC. Increased risk of lung cancer in individuals with a family history of the disease: A pooled analysis from the International Lung Cancer Consortium. Eur J Cancer. (2012) 48:1957–19682. doi: 10.1016/j.ejca.2012.01.038, PMID: 22436981 PMC3445438

[26] Ding X Chen Y Yang J Li G Niu H He R. Characteristics of familial lung cancer in yunnan-guizhou plateau of China. Front Oncol. (2018) 8:6372. doi: 10.3389/fonc.2018.00637, PMID: 30619770 PMC6305406

[27] Ang L Chan CPY Yau WP Seow WJ. Association between family history of lung cancer and lung cancer risk: a systematic review and meta-analysis. Lung Cancer. (2020) 148:129–1372. doi: 10.1016/j.lungcan.2020.08.012, PMID: 32892102

[28] Hall JM Lee MK Newman B Morrow JE Anderson LA Huey B. Linkage of early-onset familial breast cancer to chromosome 17q21. Science. (1990) 250:1684–16892. doi: 10.1126/science.2270482, PMID: 2270482

[29] Sellers TA Bailey-Wilson JE Elston RC Wilson AF Elston GZ Ooi WL. Evidence for mendelian inheritance in the pathogenesis of lung cancer. JNCI. (1990) 82:1272–12792. doi: 10.1093/jnci/82.15.1272, PMID: 2374177

[30] Lichtenstein P Holm NV Verkasalo PK Iliadou A Kaprio J Koskenvuo M. Environmental and heritable factors in the causation of cancer: Analyses of cohorts of twins from Sweden, Denmark, and Finland. N Engl J Med. (2000) 343:78–852. doi: 10.1056/NEJM200007133430201, PMID: 10891514

[31] Ryk C Kumar R Thirumaran RK Hou SM. Polymorphisms in the DNA repair genes XRCC1, APEX1, XRCC3 and NBS1, and the risk for lung cancer in never- and ever-smokers. Lung Cancer. (2006) 54:285–2922. doi: 10.1016/j.lungcan.2006.08.004, PMID: 17034901

[32] Sellers TA Ooi WL Elston RC Chen VW Bailey-wilson JE Rothschild H. Increased familial risk for non-lung cancer among relatives of lung cancer patients. Am J Epidemiol. (1987) 126:237–2462. doi: 10.1093/aje/126.2.237, PMID: 3605052

[33] Tokuhata GK Lilienfeld AM. Familial aggregation of lung cancer in humans. J Natl Cancer Inst. (1963) 30:289–3122. 13985327

[34] Koeller DR Chen R Oxnard GR. Hereditary lung cancer risk: Recent discoveries and implications for genetic counseling and testing. Curr Genet Med Rep. (2018) 6:83–882. doi: 10.1007/s40142-018-0140-2

[35] Yu HA Arcila ME Fleischut MH Stadler Z Ladanyi M Berger MF. Germline EGFR T790M mutation found in multiple members of a familial cohort. J Thorac Oncol. (2014) 9:554–5582. doi: 10.1097/JTO.0000000000000052, PMID: 24736080 PMC4412273

[36] Bailey-Wilson E Sellers TA Elston RC Evens CC Rothschild H. Evidence for a major gene effect in early-onset lung cancer. J La State Med Soc. (1993) 145:157–1622., PMID: 8486988

[37] Jin Y Xu Y Xu M Xue S. Increased risk of cancer among relatives of patients with lung cancer in China. BMC Cancer. (2005) 5:1462. doi: 10.1186/1471-2407-5-146, PMID: 16281985 PMC1299321

[38] Leung CY Huang H-L Rahman M Nomura S Abe SK Saito E. Cancer incidence attributable to tuberculosis in 2015: global, regional, and national estimates. BMC Cancer. (2020) 20:1–13. doi: 10.1186/s12885-020-06891-5, PMID: 32398031 PMC7218646

[39] Hwang SY Kim JY Lee HS Lee S Kim D Kim S. Pulmonary tuberculosis and risk of lung cancer: a systematic review and meta-analysis. J Clin Med. (2022) 11:765. doi: 10.3390/jcm11030765, PMID: 35160218 PMC8836400

[40] Su VY Yen YF Pan SW Chuang PH Feng JY Chou KT. Latent tuberculosis infection and the risk of subsequent cancer. Med (Baltimore). (2016) 95:e2352. doi: 10.1097/MD.0000000000002352, PMID: 26825880 PMC5291550

[41] Roberts T Beyers N Aguirre A Walzl G. Immunosuppression during active tuberculosis is characterized by decreased interferon-γ production and CD25 expression with elevated forkhead box P3, transforming growth factor-β, and interleukin-4 mRNA levels. J Infect Dis. (2007) 195:870–8. doi: 10.1086/511277, PMID: 17299718

[42] da Silva AL Bresciani MJ Karnopp TE Weber AF Ellwanger JH Henriques JA. DNA damage and cellular abnormalities in tuberculosis, lung cancer and chronic obstructive pulmonary disease. Multidiscip Respir Med. (2015) 10:38. doi: 10.1186/s40248-015-0034-z, PMID: 26688728 PMC4684909

[43] Stabile LP Davis AL Gubish CT Hopkins TM Luketich JD Christie N. Human non-small cell lung tumors and cells derived from normal lung express both estrogen receptor alpha and beta and show biological responses to estrogen. Cancer Res. (2002) 62:2141–50., PMID: 11929836

[44] Mollerup S Jorgensen K Berge G Haugen A. Expression of estrogen receptors alpha and beta in human lung tissue and cell lines. Lung Cancer. (2002) 37:153–9. doi: 10.1016/s0169-5002(02)00039-9, PMID: 12140138

[45] Yin X Kishida R Abe SK Islam MR Rahman MS Saito E. Association between reproductive factors with lung cancer incidence and mortality: A pooled analysis of over 308,000 females in the Asia cohort consortium. Int J Cancer. (2024) 154:2090–105. doi: 10.1002/ijc.34866, PMID: 38375919

[46] Yin X Zhu Z Hosgood HD Lan Q Sewo WJ. Reproductive factors and lung cancer risk: a comprehensive systematic review and meta-analysis. BMC Public Health. (2020) 20:1458. doi: 10.1186/s12889-020-09530-7, PMID: 32977782 PMC7519481

[47] Rosell R Moran T Queralt C Porta R Cardenal F Camps C. Screening for epidermal growth factor receptor mutations in lung cancer. N Engl J Med. (2009) 361:958–67. doi: 10.1056/NEJMoa0904554, PMID: 19692684

[48] Schwartz AG Wenzlaff AS Prysak GM Murphy V Cote ML Brooks SC. Reproductive factors, hormone use, estrogen receptor expression and risk of non-small-cell lung cancer in women. J Clin Oncol. (2007) 25:5785–92. doi: 10.1200/JCO.2007.13.3975, PMID: 18089876

[49] Taioli E Wynder EL. Re: Endocrine factors and adenocarcinoma of the lung in women. J Natl Cancer Inst. (1994) 86:869–70. doi: 10.1093/jnci/86.11.869, PMID: 8182770

[50] Seng ZB Jue WT Di ZQ Peng G Wu JM. Risk factors analysis of lung adenocarcinoma in women. Chin J Public Health. (2000) 16:536–9.

[51] Liu B Quan X Xu C Lv J Li C Dong L. Lung cancer in young adults aged 35 years or younger: A full-scale analysis and review. J Cancer. (2019) 10:3553–35593. doi: 10.7150/jca.27490, PMID: 31293660 PMC6603399

[52] Xie S Hu Q Wu Z Wang B He Y Huang Q. Clinical and genetic characteristics of early-onset lung adenocarcinoma in a large chinese cohort. Lung. (2025) 119(6):1196–204. doi: 10.1016/j.athoracsur.2024.09.014, PMID: 39313087

[53] Zhang J Chen SF Zhen Y Xiang J Wu C Bao P. Multicenter analysis of lung cancer patients younger than 45 years in Shanghai. Cancer. (2010) 116:3656–36623. doi: 10.1002/cncr.25100, PMID: 20564076

[54] Tanaka K Hida T Oya Y Yoshida T Shimizu J Mizuno T. Unique prevalence of oncogenic genetic alterations in young patients with lung adenocarcinoma. Cancer. (2017) 123:1731–17403. doi: 10.1002/cncr.30539, PMID: 28177518

[55] Malik PS Pathak N Sharma A Birla M Rastogi A Sharma A. Young onset lung cancer in India: insights into clinical, demographic, and genomic profiles. Clin Lung Cancer. (2025) 26(5):420–8.e4. doi: 10.1016/j.cllc.2025.02.014, PMID: 40122770

[56] Sacher AG Dahlberg SE Heng J Mach S Jänne PA Oxnard GR. Association between younger age and targetable genomic alterations and prognosis in non-small-cell lung cancer. JAMA Oncol. (2016) 2:313–3203. doi: 10.1001/jamaoncol.2015.4482, PMID: 26720421 PMC4819418

[57] Luo W Tian P Wang Y Xu H Chen L Tang C. Characteristics of genomic alterations of lung adenocarcinoma in young never-smokers. Int J Cancer. (2018) 143:1696–17053. doi: 10.1002/ijc.31542, PMID: 29667179 PMC6175072

[58] Pan X Lv T Zhang F Fan H Liu H Song Y. Frequent genomic alterations and better prognosis among young patients with non-small-cell lung cancer aged 40 years or younger. Clin Transl Oncol. (2018) 20:1168–11743. doi: 10.1007/s12094-018-1838-z, PMID: 29460035

[59] Ren W Zhang B Ma J Li W Lan J Men H. EML4-ALK translocation is associated with early onset of disease and other clinicopathological features in Chinese female never-smokers with non-small-cell lung cancer. Oncol Lett. (2015) 10:3385–33923. doi: 10.3892/ol.2015.3740, PMID: 26788139 PMC4665761

[60] Hou H Zhu H Zhao H Yan W Wang Y Jiang M. Comprehensive molecular characterization of young chinese patients with lung adenocarcinoma identified a distinctive genetic profile. Oncologist. (2018) 23:1008–10153. doi: 10.1634/theoncologist.2017-0629, PMID: 29700208 PMC6192606

[61] Nagashima O Ohashi R Yoshioka Y Inagaki A Tajima M Koinuma Y. High prevalence of gene abnormalities in young patients with lung cancer. J Thorac Dis. (2013) 5:27–303. doi: 10.3978/j.issn.2072-1439.2012.12.02, PMID: 23372947 PMC3547997

[62] Gitlitz BJ Morosini D Sable HA Addario B Jennings MB Mach S. The genomics of young lung cancer study [abstract. J Clin Oncol. (2016) 34.

[63] Tian Y Ma R Zhao W Wang S Zhou C Wu W. Comprehensive characterization of early-onset lung cancer, in Chinese young adults. Nat Commun. (2025) 16:19763. doi: 10.1038/s41467-025-57309-4, PMID: 40000630 PMC11861273

[64] Cai L Chen Y Tong X Wu X Bao H Shao Y. The genomic landscape of young and old lung cancer patients highlights age-dependent mutation frequencies and clinical actionability in young patients. Int J Cancer. (2021) 149:883–8923. doi: 10.1002/ijc.33583, PMID: 33811322

[65] Hollingsworth MA Swanson BJ. Mucins in cancer: protection and control of the cell surface. Nat Rev Cancer. (2004) 4:45–603. doi: 10.1038/nrc1251, PMID: 14681689

[66] Das S Batra SK. Understanding the unique attributes of MUC16 (CA125): potential implications in targeted therapy. Cancer Res. (2015) 75:4669–46743. doi: 10.1158/0008-5472.CAN-15-1050, PMID: 26527287 PMC4651718

[67] Kufe DW. Mucins in cancer: function, prognosis and therapy. Nat Rev Cancer. (2009) 9:874–8853. doi: 10.1038/nrc2761, PMID: 19935676 PMC2951677

[68] Das S Rachagani S Torres-Gonzalez MP Lakshmanan I Majhi PD Smith LM. Carboxyl terminal domain of MUC16 imparts tumorigenic and metastatic functions through nuclear translocation of JAK2 to pancreatic cancer cells. Oncotarget. (2015) 6:5772–57873. doi: 10.18632/oncotarget.3308, PMID: 25691062 PMC4467401

[69] Thériault C Pinard M Comamala M Migneault M Beaudin J Matte I. MUC16 (CA125) regulates epithelial ovarian cancer cell growth, tumorigenesis and metastasis. Gynecol Oncol. (2011) 121:434–4433. doi: 10.1016/j.ygyno.2011.02.020, PMID: 21421261

[70] Lakshmanan I Ponnusamy MP Das S Chakraborty S Haridas D Mukhopadhyay P. MUC16 induced rapid G2/M transition via interactions with JAK2 for increased proliferation and anti-apoptosis in breast cancer cells. Oncogene. (2012) 31:805–8563. doi: 10.1038/onc.2011.297, PMID: 21785467 PMC3288594

[71] Aithal A Rauth S Kshirsagar P Shah A Lakshmanan I Junker WM. MUC16 as a novel target for cancer therapy. Expert Opin Ther Targets. (2018) 22:675–6863. doi: 10.1080/14728222.2018.1498845, PMID: 29999426 PMC6300140

[72] Chen Y Huang Y Kanwal M Li G Yang J Niu H. MUC16 in non-small cell lung cancer patients affected by familial lung cancer and indoor air pollution: clinical characteristics and cell behaviors. Transl Lung Cancer Res. (2019) 8:476–4884. doi: 10.21037/tlcr.2019.07.10, PMID: 31555520 PMC6749135

[73] Dal Bello MG Alama A Coco S Vanni I Grossi F. Understanding the checkpoint blockade in lung cancer immunotherapy. Drug Discov Today. (2017) 22:1266–73. doi: 10.1016/j.drudis.2017.05.016, PMID: 28600190

